# Biofilm Formation by Pathogenic Bacteria: Applying a *Staphylococcus aureus* Model to Appraise Potential Targets for Therapeutic Intervention

**DOI:** 10.3390/pathogens11040388

**Published:** 2022-03-23

**Authors:** Zahra Sedarat, Andrew W. Taylor-Robinson

**Affiliations:** 1 Cellular & Molecular Research Centre, Shahrekord University of Medical Sciences, Shahrekord, Iran; sedaratzahra@gmail.com; 2 College of Health Sciences, Vin University, Gia Lam District, Hanoi, Vietnam; 3 Center for Global Health, Perelman School of Medicine, University of Pennsylvania, Philadelphia, PA 19104, USA

**Keywords:** pathogen, bacteria, *Staphylococcus*, infection, biofilm, treatment, antibiotic, antimicrobial

## Abstract

Carried in the nasal passages by up to 30% of humans, *Staphylococcus aureus* is recognized to be a successful opportunistic pathogen. It is a frequent cause of infections of the upper respiratory tract, including sinusitis, and of the skin, typically abscesses, as well as of food poisoning and medical device contamination. The antimicrobial resistance of such, often chronic, health conditions is underpinned by the unique structure of bacterial biofilm, which is the focus of increasing research to try to overcome this serious public health challenge. Due to the protective barrier of an exopolysaccharide matrix, bacteria that are embedded within biofilm are highly resistant both to an infected individual’s immune response and to any treating antibiotics. An in-depth appraisal of the stepwise progression of biofilm formation by *S. aureus*, used as a model infection for all cases of bacterial antibiotic resistance, has enhanced understanding of this complicated microscopic structure and served to highlight possible intervention targets for both patient cure and community infection control. While antibiotic therapy offers a practical means of treatment and prevention, the most favorable results are achieved in combination with other methods. This review provides an overview of *S. aureus* biofilm development, outlines the current range of anti-biofilm agents that are used against each stage and summarizes their relative merits.

## 1. Introduction

Biofilm is a complicated bacterial structure that was recognized for the first time by the Dutch microscopist Anton Van Leeuwenhoek in dental plaque during the 1670s. Until around 50 years ago, very few studies had been performed on biofilm properties. Following the invention of the electron microscopy, it was revealed that biofilm is a microbial community composed of bacteria [1,2]. Within this unique structure microorganisms possess multicellular behavior that is distinct from that of simple planktonic cells, and they are typically at least 500 times more resistant to antibacterial agents [3]. This multicellular environment is beneficial to bacterial survival for extended periods and is thus considered a self-defense measure to safeguard against unfavorable conditions. Advantages include colonization under suitable conditions, establishment of a community in which there is cooperation, and production of biofilm as a default growth state to compete with hostile circumstances [4,5]. The structural complexity of a biofilm is founded on its constituent sugar types, and it is these that determine virulence [2,6]. In this community, cell density is comparatively high, ranging between 10^8^ to 10^11^ cells per gram. As a result, biofilm production is thought to account for over 80% of persistent clinical infections [3]. A property of multiple species of bacteria related to biofilm synthesis is the ability to attach to numerous living and inanimate surfaces. These include natural rock, lung tissue, intestinal tissue, tooth enamel, urinary catheters, vascular access devices, endotracheal tubes, tracheostomies, enteral feeding tubes, wound drains, and other medical devices [7,8].

*Staphylococcus aureus* is considered a principal cause of nosocomial infections, which are a major burden to healthcare systems globally. This typically commensal Gram-positive bacterium is a leading source of opportunistic infections including those relating to skin, osteoarticular pathology, endocarditis, and contaminated introduced devices [9]. Medical implants and host tissues can be covered by this bacterium, when the biofilm so formed plays a pivotal role in chronic, difficult-to-treat infections. *S. aureus* is equipped with various virulence factors including enzymes, extracellular toxins, clumping factors and surface proteins [10]. Surface attachment to a substrate triggers the formation of biofilm, which provides a physical barrier that is difficult to penetrate. The enclosed environment can drive chronic infection in which the bacterial community is resistant to antibacterial agents and to host immunity. Hence, bacteria within biofilm are typically more resistant to antibiotics than are planktonic cells [11,12]. Typically, the prevalence of methicillin-resistant *S. aureus* (MRSA) in clinical specimens is closely associated with a potent ability to produce biofilm. For instance, in a recent study from Nepal most MRSA isolates formed biofilm [13].

Another opportunistic pathogen that can form biofilm, *Pseudomonas aeruginosa* is responsible for several disorders including respiratory tract infections. Cystic fibrosis is a health-threatening condition that is a consequence of persistent lung infection with *P. aeruginosa* [14]. *Streptococcus mutans*, *Escherichia coli* and *Enterococcus faecalis*, *Salmonella enterica* serovars, *Klebsiella pneumoniae*, *Listeria monocytogenes*, *Bacillus subtilis* and *Helicobacter pylori* are responsible for other biofilm-driven infections including periodontitis, urinary tract infections, gastroenteritis, food-borne illness, indwelling device infections and gastrointestinal disorders, respectively [15,16,17,18,19,20]. The prevalence of these long-served infections is considerable. According to data from the US National Institutes of Health, 65% of all microbial infections and 85% of chronic infections are attributed to biofilm formation [1]. The rate of indwelling device infections is thought to range from 2% to 10%, and the highest rate of infection, at 40%, is associated with ventricular-assisted devices [21].

The complex structure of biofilm promotes long-term infections via various pathways. High mutation rates, numerous virulence factors, slow growth rates and adaptability are each ascribed to *P. aeruginosa* in causing cystic fibrosis. Antimicrobial resistance (AMR) genes, including those encoding enzymes that confer resistance to β-lactam and aminoglycoside antibiotics, as well as multi-drug efflux pumps, which present a major obstacle for therapy are exaggerated in those bacterial species that live in biofilm. Hence, the effective treatment of infections by *S. aureus*, *Staphylococcus epidermidis*, *P. aeruginosa* and other bacteria that share such features has become a formidable ongoing challenge [14,22].

An important issue is heterogeneity of bacteria. The composition of biofilm is dependent not only on environmental conditions, but also on microorganism diversity. The medical challenge that is presented is the outcome of the production of extracellular polymeric substances (EPS) by a wide range of microorganisms that form biofilm, which results in phenotypic heterogeneity, as well as interactions between different species competing to occupy the same ecological niche. Another feature of biofilm is its varying physiochemical properties from surface to interior. Among those bacteria located in the superficial layer, regeneration take places. So-called “persister” cells, which exist in various bacteria, are distinct from active cells. These non-growing dormant cells are metabolically inactive and thus tolerant to antibiotics. While comprising only a small proportion of the total cell population, they can remain even after antibiotic concentrations drop [23,24,25,26]. Additionally, small colony variants (SCVs), a subpopulation of many species of bacteria, emerge due to mutation in response to harsh conditions. These slow-growing variants are unstable and so can revert to their normal phenotype. Higher rates of antibiotic resistance by SCVs are implicated in infectious disease chronicity [27].

In summary, there is growing public health concern surrounding the increasing prevalence of AMR, the pressing requirement for this to be controlled and the need for novel antibacterial agents. In this context, a multinational program was instigated recently in order to take authoritative action to safeguard against this threat [28]. This article reviews the main drivers and mechanisms of biofilm formation as well as, informed by this knowledge, considering the new strategies being developed to combat biofilm-forming bacteria. The focus is on *S. aureus* as the most common cause of biofilm formation of public health relevance and that which is the most studied. The principles of this model infection may be applied to other pathogenic biofilm-forming bacteria that contribute to the increasing global challenge of AMR.

## 2. Biofilm Formation

There are several prerequisites to form biofilm, the most crucial of which is a suitable substrate. The nature and condition of the surface are key determinants. Bacterial colonization occurs more frequently and at a greater rate on rough surfaces. This means that distinctive materials such as metal, glass and Teflon have different potential for biofilm development. Similarly, the rate and extent of adherence vary dependent upon the composition of chemicals that coat the biofilm [8].

A major influence on biofilm formation is the environment in which bacteria exist. A pivotal factor is the oxygen and nutrient gradient. Nutrient-deficient conditions can trigger stressed bacteria to form this complex structure, within which they are able to withstand the hostile surroundings. By responding to a given stress level by forming biofilm or not, cells determine their own fate. They do this by producing an extracellular matrix (ECM) by which to cover the multicellular aggregation, whereupon they can survive much longer when exposed to the host immune system or to antibiotics [29,30,31,32].

While the structural complexity of biofilm enables bacterial growth on numerous surfaces, from a medical perspective, artificial devices can provide fertile ground for it to become established. Based on studies over several years, different stages of biofilm formation are defined, including attachment, maturation and detachment/dispersal [33,34,35,36,37]. In order to target biofilm for effective treatment an in-depth knowledge is required of the stages of its formation, as well as an understanding of the unique structure of the ECM and of quorum sensing, the ability of bacteria to detect and to respond to changes in cell population density through gene regulation [38]. In the following sections we provide details primarily using *S. aureus* as a biofilm study model.

### 2.1. Microbial Surface Adhesion

**Figure 1:**
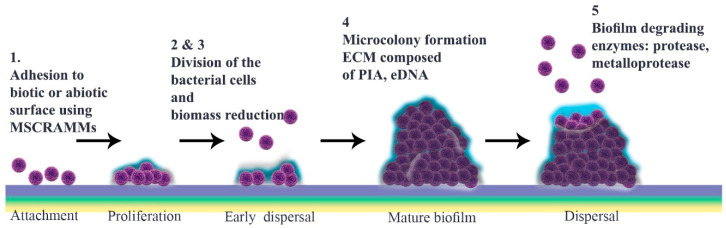
Schematic representation of *Staphylococcus aureus* biofilm development. This is divided chronologically into a four-step progression: (**1**) attachment; (**2**) multiplication; (**3**) maturation; and (**4**) detachment. First, bacteria adhere to different substrates, including biomaterial surfaces and host tissues, by using cell–cell interactions and their virulence factors such as surface proteins. Gradually, attached bacteria start to divide and proliferate. Many antimicrobial agents target this metabolically active multiplication stage. Among these, nanoparticles, bacteriophages, antibodies, phytochemicals, and enzymes are noteworthy (Table 1). The maturation stage follows, during which a mature biofilm is formed. At this point, a mass of accumulated bacteria is surrounded by an exopolysaccharide matrix. Laser shock or photodynamic therapy can attack this outer surface (Table 1). Finally, during the detachment stage, physical forces, and enzymes such as proteases, as well as quorum sensing system, promote the release of daughter bacterial cells (**5**). This stage is targeted by most classes of antibiotic.

Initial steps towards biofilm formation involve attachment to uncoated or coated surfaces, utilizing cell wall anchors as required by the nature of the underlying biotic or abiotic substrate [39] (Figure 1). Bacteria can adhere to each other or to solid surfaces as well as to interfaces of either solid/liquid, liquid/air, liquid/liquid or solid/air. They perform this action using their flagella, pili, or fimbriae [6,40]. Non-motile bacteria, exemplified by staphylococci, can attach to abiotic surfaces passively.

Microbial surface components recognizing adhesive matrix molecules (MSCRAMMs), adhesin proteins that are utilized by staphylococci, mediate attachment to medical devices [41]. Members of the MSCRAMM family include protein A, as well as fibronectin-binding, serine-aspartate repeat, clumping factor and collagen adhesion proteins, biofilm-associated protein, and *S. aureus* surface proteins (FnBP, Sdr, ClfB, Bap and SasG) [42,43,44,45,46,47,48]. Under specific circumstances electrostatic and hydrophobic interactions may also play a significant role. In addition, negatively charged teichoic acids (TAs) and major autolysin enable attachment [49,50,51]. During this multiplication stage, bacteria may not be sufficiently stable, so, in order to survive, the immature biofilm adopts strategies such as producing different factors—including *S. aureus* surface proteins—which help biofilm formation and its accumulation [52].

Several strategies aim to prevent development of biofilm at this early stage. Potential targets include bacterial interactions with surfaces and their receptors such as fibrinogen and fibronectin. Preventing attachment can either inhibit adhesion or bacterial growth. As TAs contain D-alanine residues, the surface of *S. aureus* is negatively charged, which is a pivotal point in the initial adhesion process. Negatively charged implant devices provide repulsive forces, thereby disabling adhesion. Furthermore, there is a range of antimicrobial chemicals and features, including antibacterial and antiadhesion coatings, which are mentioned below [50,52,53,54,55].

### 2.2. Development to Mature Biofilm

What are the main features required for a biofilm to mature? Prior to microcolony formation, *S. aureus* cell attachment is followed by a dispersal stage that is independent of final detachment. The initial proliferation that takes place during biofilm formation requires strengthening intercellular binding, which involves various virulence factors including MSCRAMMs, SasG, Bap and protein A. Once cells multiply, they start to disseminate, a stage that is defined as “early dispersal”, in order to restructure the biofilm. This process is aided by nucleases. Microcolonies, which are characteristic of “mature biofilm”, form only after biomass reduction. Bacterial vulnerability results in multiplication as a strategy to enhance cell interactions prior to the “exodus” stage, the role of which is not yet elucidated [56,57,58,59,60,61,62].

The most notable structural component of biofilm is ECM, in which bacterial cells embed. This comprises polymeric molecules secreted from daughter cells composed of proteins, polysaccharide-intercellular adhesins (PIA) and/or extracellular DNA (eDNA). During multiplication, cells are protease-sensitive, indicative of the fact that ECM is composed mostly of protein components like those that bind to eDNA to stabilize this “early biofilm”. These proteins are degraded by nuclease enzymes secreted from bacterial cells at the early dispersal stage. At this time, a protein/DNA-based ECM is predominant [56,63].

Among polymeric molecules involved in ECM, PIA (also known as poly-N-acetylglucosamine; PNAG), is characteristic of *Staphylococcus* and has a cationic nature that can facilitate attachment [57,59,64]. Enzymes encoded by the ica operon catalyze production of PIA. While this operon exists in most *S. aureus* isolates, its expression is affected by levels of glucose, anaerobicity, osmotic stress and CO_2_. PIA increases biofilm retention and its resistance to antimicrobial peptides (AMPs) through deacetylation. Additionally, it is believed that the ica operon is under phase variation, which has a role in slipped strand mispairing and leads to an on/off switch for expression of the products [65,66,67].

In the subsequent stage, a three-dimensional “mature biofilm” forms. This has two towers either side of a central channel [68]. Different models are described for “microcolony” formation, which is a cue for maturation. Mature biofilm has a diverse and metabolically distinct structure that makes it resistant to unwanted environmental and stressful drivers. Interestingly, distinct gene patterns are responsible for coding these microcolonies at different rates [57,69,70]. EPS, which contain several components including polysaccharides, glycolipids, protein, glycoproteins, PIA and eDNA, are thought to constitute around 90% of the microcolony structure [71,72,73]. Inside this, not only can bacteria exchange nutrients and waste but they can also be dispersed over far distances [34,72]. Through phenol-soluble modulin (PSM)-mediated dispersal, alpha-helical peptides can break up channels from thick biofilm cells or from those cells belonging to different foci in the basal layer that have remained after the so-called ‘exodus’ [69] (Figure 1).

Treatment of an infected individual during this mature stage is extremely challenging, as it is the most stable form of biofilm [74]. It presents several recognized barriers to the effective action of antibiotics. The EPS matrix can reduce antibiotic efficiency by providing an obstacle to diffusion and a storage for enzymes. This natural defense can lessen phage recognition that depends on EPS. This is an important consideration when determining treatment targets. Similarly, eDNA can diminish antibiotic performance by bolstering the cellular structure. Quorum sensing, a distinctive feature of biofilm, controls production of virulence factors and thereby promotes antimicrobial resistance. Persister cells offer another potential therapeutic target [23,26,75,76]. Antibodies can either target MSCRAMMs to prevent attachment, or cover host cell surfaces to heighten clearance of bacteria. Regarding vaccine design against biofilm-producing bacteria, PIA is a potential target [77]. ClfA, ClfB, FnBPA and FnBPB are also good candidate antigens as their expression is ubiquitous among *S. aureus* strains and each participates in biofilm formation [78,79].

Physical removal by surgery and debridement for currently embedded bacteria, antibiotic regimens and application of ECM-degrading enzymes are notable therapies [80,81,82]. Although justified experimentally, these methods are not entirely practical to translate to large-scale clinical use. In some cases, antibiotic therapy should follow physical approaches to enhance efficacy because bacteria embedded in biofilm are more resistant than planktonic cells [83,84].

### 2.3. Detachment

There are several proposals to explain how biofilm is dispersed (Figure 1), including isolating new cells from growing ones, reducing biofilm mass, quorum sensing and triggering by insufficient nutrient levels [85]. It is thought that matrix composition determines physical forces that can propel this stage through erosion, sloughing or abrasion [8]. Different enzymes, typically proteases, can weaken protein-dependent biofilm and thereby facilitate its degradation. *S. aureus* and *S. epidermidis* produce various proteases including serine/cysteine protease and metalloprotease [86]. Similarly, nuclease and nuclease 2 (NUC and NUC2) play important roles by disrupting neutrophils and altering biofilm formation as well as targeting eDNA in the matrix [58,87,88]. Furthermore, P3 promoter expression in accessory gene regulator (*agr*) quorum sensing has a function in detachment of cells, which can initiate dispersal by autoinducer peptide addition or through glucose depletion. Production of proteases is under the control of the *agr* quorum sensing system, following activation of which autoinducing peptides (AIPs) are detectable, implicating the next stage as dispersal [89,90,91,92].

Regarding treatment, antibiotic efficacies have increased by using enzymes as dispersal agents. In terms of prevention, utilizing dispersal agents for pretreatment of medical devices not only suppresses proliferation, but also facilitates biofilm purgation. While these appear to be promising therapeutic advances, some concerns have been expressed. For instance, a chronic infection occurs if the administered dose of some antibiotics is unable to permeate the biofilm as sub-inhibitory concentrations can drive *agr* activation or eDNA release. Moreover, embolism as a consequence of degrading matrix components is a possible adverse reaction [11].

### 2.4. Quorum Sensing

Quorum sensing plays a substantial role during different stages of biofilm formation including attachment and detachment. This cell-to-cell signaling is under the control of *agr* quorum sensing or an accessory gene regulator [69,93,94,95]. There are four loci, namely *agr D*, *agr B*, *agr C*, *agr A*, which encode the central system and between each of which there is a close relationship. *S. aureus* has only one copy of each locus, but this is not proven for other species. Via this system, bacteria communicate by producing hormone-like AIPs. Once the rate of signal generation reaches a threshold level signal transduction is activated. The fluctuation in cell density provides the main stimulus for this gene regulation. Bacterial AIPs are responsible for such key activities as biofilm formation, antibiotic resistance, conjugation, and virulence. Therefore, quorum sensing is considered a potential target for therapy and infection control [95,96]. Different types of quorum sensing are used by Gram-positive and Gram-negative bacteria or are common to both [97,98].

Although results from *in vitro* studies are not altogether consistent the consensus view is that quorum sensing is a requirement for biofilm formation and that detachment is controlled at the AIP level. Not only does the *agr* system propel detachment of *S. aureus* by adding AIP or glucose to a mature biofilm, it is also necessary to suppress biofilm. In one study, 78% of *S. aureus* that formed biofilm was *agr*-negative. Such findings strengthen the argument that the quorum sensing system may be harnessed as a biofilm blocker. Moreover, it seems that proteases, an important propeller in biofilm dispersal, are under *agr* regulation [89,90,94,99,100].

## 3. Anti-Biofilm Treatments

### 3.1. Antibiotics in Single and Combination Therapy

Antibiotics can be used both as prevention and therapy. In terms of current treatments, different strategies include raising dosage concentrations and combining therapy with other antimicrobial agents [101]. The maturity of the mass of a biofilm should be considered as mature and therefore as less susceptible to treatment [102,103]. This applies to a wide range of species of both facultative aerobic and facultative anaerobic bacteria that form biofilm. These include the classically non-motile Gram-positive *S. aureus*, *S. epidermidis*, *Enterococcus faecium* and Gram-negative *Acinetobacter baumannii* and *Klebsiella pneumoniae*, as well as the flagellated Gram-negative *P. aeruginosa* and *Enterobacter* spp. [104,105]. In selecting a suitable antibiotic sufficient biofilm penetration is an important consideration. Hence, tetracyclines, macrolides, rifamycins, lincosamides, quinolones, fusidic acid, oxazolidinones, sulfonamides and nitroimidazole are preferred to glycopeptides, aminoglycosides, polymyxins and β-lactamases as they have the capability to penetrate deeper [106]. In addition to biofilm age and level of resistance to a given antibiotic, broader considerations for treatment include appropriate duration of antibiotic regimen and dosage optimization [107].

There are several tolerance mechanisms utilized by bacteria that enable them to show resistance and persistence in the face of antibiotic treatment. A growing concern surrounds the fact that biofilms are not only resistant to antibiotics, but frequently also to the host immune response [108]. In order to combat the thorny problem of antibiotic resistance, suggested solutions include gaining a deeper knowledge of phenotypic and genotypic characteristic features of biofilm [109]. Mounting evidence indicates that acquiring resistant genes via genetic exchange and through EPS plays a pivotal role in antibacterial tolerance [110,111].

A specific feature of biofilm is ‘recalcitrance’, a term used to describe its capability to survive in the presence of high doses of antibiotics [112]. Bacteria within biofilm can exhibit resistance to multiple treatments, even in the presence of high concentrations of bactericidal and bacteriostatic antibiotics and toxic compounds, in stark contrast to their planktonic existence. Noteworthy among various mechanisms by which this complex phenomenon may occur are antibiotic efflux, enzyme activity and reduced permeability. Minimum inhibitory concentration (MIC) can be used as a quantitative measure of antibiotic resistance; the higher the MIC, the more resistant. Resistance and tolerance each has a potential role in biofilm recalcitrance. Exposure to both bacteriostatic and bactericidal antibiotics can lead to resistance, while it is only the use of bactericidal antibiotics that may result in tolerance [112,113,114].

The MIC and minimum bactericidal concentration (MBC) are the lowest levels of an antimicrobial agent, typically an antibiotic, required to prevent visible growth upon overnight incubation (i.e., to cause cell stasis) and to kill a particular bacterium, respectively [115]. Similarly, MBIC and MBEC refer to minimum biofilm inhibitory concentration and minimum biofilm eradication concentration, respectively [116]. MIC is much higher for those bacteria that form biofilm compared to those than do not [117]. This concurs with the observation that biofilms are resistant to antibiotics concentrations up to 1000 × greater than those required to kill free-living bacteria [101], which signifies a pressing need to use combination therapy instead of monotherapy. The emergence of *S. aureus* isolates that are resistant to multiple antibiotics is a real concern, especially as it is exaggerated among MRSA strains [118,119].

Performing antibiotic sensitivity tests is necessary to select an appropriate choice and dose of treatment. Determination of MIC and MBEC of bacteria can inform tailored treatments and help to reduce the spread of resistant strains. *Staphylococcal* isolates from biofilm show a much higher breakpoint for MBEC than for MIC, indicating the importance of applying both biofilm susceptibility tests [120]. While vancomycin MBEC and MIC of planktonic cells are similar, for biofilm-producing isolates they are markedly different, so from a clinical perspective MBEC is the preferred measure [121]. Despite the availability of standardized methods to treat biofilm, most successful approaches were determined on planktonic cells. Although MBEC and MBIC values are proposed, this is confounded by limited evidence and complexity of correlation between innate activity towards planktonic cells and those in biofilm [122].

Multi-drug resistance has become common among MRSA strains. The formerly frontline β-lactam antibiotic methicillin targets penicillin-binding proteins (PBPs), enzymes that are essential to peptidoglycan synthesis. Yet, due to genetic mutation under the selective pressure imposed by overuse, PBP and PBP2a have become principal resistance factors [123,124]. One study from Nepal showed that the vast majority of multi-drug resistant isolates are MRSA with potential to produce biofilm [13]. Similarly, all strains of MRSA from nasal carriers possessed the capacity to form a biofilm that showed resistance to multiple antibiotics [125]. However, another study reported no difference between methicillin-sensitive *S. aureus* and MRSA strains to form biofilm [105], implying that there is no direct correlation between the ability of an isolate to form biofilm and its pattern of antibiotic resistance.

A currently largely successful *S. aureus* anti-biofilm agent is the glycopeptide antibiotic vancomycin, which acts by interrupting cell wall synthesis [126,127]. Vancomycin is the preferred treatment for MRSA at present, although recently vancomycin-resistant *S. aureus* (VRSA) has been reported. The emergence of these strains, a major public health concern, could be for one or more reasons. Vancomycin is a large compound, which can lead to its weak penetration of biofilm. Additionally, as it inhibits oxygen and nutrient uptake [128]. In order to address this issue, combination therapy with antibiotics like rifampin and linezolid is proposed [129,130,131,132]. Many tested strains of *S. aureus* and most of *S. epidermidis* are susceptible to rifampin, which can penetrate biofilm [133,134]. In keeping with this, rifampin was shown to be the only good candidate for biofilm therapy in isolates with relatively high MBEC for each of vancomycin, rifampin and gentamicin [135]. The efficacy of rifampin in combination with vancomycin is due to reducing bacterial adhesion [136]. On a cautionary note, as resistance to rifampin is acquired rapidly it should not be used alone [137].

The lipopeptide antibiotic daptomycin, an alternative treatment option for MRSA and VRSA, effectively targets biofilm [138]. Both rifampin and daptomycin can disrupt MRSA biofilm at lower concentrations than that of tigecycline required to eradicate mature biofilm. Other antibiotics are able only to prevent cell attachment [139].

In some cases, antibiotic therapy may not be completely successful due to low permeability to the biofilm matrix [35]. When this occurs, either removal of the foreign body, long-term single antibiotic treatment at high dosage and/or combination therapy is advised. Furthermore, in response to a substantial increase in reports of MRSA and VRSA in recent years, a range of modern medical technologies, such as laser therapy and nanoparticles, have been investigated in attempts to enhance antibiotic efficacy. There are several benefits of harnessing nanoparticles including their high surface area to volume ratio, capacity for drug transportation and antibiotics protection against exposure to pH and enzymes, each of which enhances the efficacy of an administered antibiotic [106,140,141]. When gold nanoparticles were used alongside laser therapy to combat resistant strains of *S. aureus* and *P. aeruginosa* biofilm viability reduced and, conversely, antibiotic sensitivity increased [142]. In another study in which gold nanoparticles were conjugated to antibody specific to *S. aureus* peptidoglycan and activated by exposure to laser, bacterial cell counts were substantially reduced [123]; potentially, such technology could be used in tandem with antibiotics to boost their efficacy. Continuing research is exploring how to effectively harness enzymes as anti-biofilm agents. Enzymatic degradation is a potentially suitable replacement to using toxic compounds to facilitate antibiotic penetration of biofilm. For example, *Mycobacterium* proteases have shown promise [143].

In order to combat the global health crisis of escalating antibiotic resistance, guidelines on responsible antimicrobial stewardship are urgently required. Yet, currently there is no international consensus. Tacking discrepancies that may arise when implementing novel antibiotics is critical to their longevity of use. A wide range of previously heavily used antibiotics is no longer effective due to elevated MBC and MIC doses. Thus, carefully applying alternative treatments is a pressing therapeutic need [144,145].

### 3.2. Other Anti-Biofilm Agents

*Vaccines and Antibodies:* Ongoing research aims to identify a suitable vaccine candidate to prevent *S. aureus* biofilm-related infections, which has served to highlight the emergence of antibiotic-resistant strains. Although preliminary results have shown promise, a potential candidate has yet to reach advanced stages of development. Examples can be seen in experimental vaccines against *S. aureus* iron surface determinant B (IsdB), PIA, FnBP and ClfB, all of which fail to target biofilm [77,146,147]. Unfortunately, most of these constructs that target capsular polysaccharides have stalled in the phase II clinical trial as they do not elicit sufficient protective immunity. Nonetheless, their capacity to ameliorate biofilm conditions can be improved by pairing with Freund’s adjuvant [148]. Similarly, conjugating PIA with diphtheria toxoid produces a strong adjuvant effect. Pre-clinical *in vivo* trials on PIA-based constructs showed promise [77]. Not all clinical isolates, however, express these virulence factors. Evidently, anti-biofilm immunization shows early potential, but requires further research.

Antibody-based approaches are another promising way to overcome biofilm. These act at several different levels including attachment and targeting mature biofilm. Many attempts to treat bacterial infections using antibodies have targeted biofilm. TRL1068 was designed against DNABII epitope, an eDNA, with promising results [149]. Likewise, polyclonal antibodies tested against PhnD antigen showed an ability to inhibit biofilm development by both *S. aureus* and *S. epidermidis* [78,150]. Monoclonal antibodies to FnBP and ClfA, when combined with antibodies against the membrane-disrupting alpha-toxin, prevented biofilm formation. The antibody targets FnBPA, SasG, Atl and Atl-Amd have been tested only *in vitro*, while ClfA, Can and Atl-Gmd have undergone *in vivo* trials with satisfactory results [78]. It is critical to consider precautions when designing passive *S. aureus* vaccines. Of note are the presence of multiple *S. aureus* virulence factors, knowledge gaps surrounding immunity against *S. aureus* and the need for information from human trials [55,151,152].

*Biofilm-degrading enzymes:* Dispersin B is an enzyme that is produced by *Aggregatibacter actinomycetemcomitans.* It degrades mature biofilm and thus may provide a novel therapy [84]. Similarly, rhDNase has a potent effect on eDNA and so could be exploited to either prevent or treat infection. Additionally, it increases the sensitivity of biofilm to antibiotics such as tobramycin. Dispersin B shows similar biocidal properties towards biofilm when paired with tigecycline or vancomycin [153]. Moreover, dispersin B can act alongside proteases to improve treatment outcomes [154].

*Probiotics:* Microorganisms that live beneficially within the human host’s body are described as “probiotic”, a term particularly ascribed to commensal gut microflora. They can interfere with potentially pathogenic bacterial growth through disrupting the biofilm community by competitively inhibiting attachment to shared substrates. Probiotics are a preferred choice to eradicate biofilm-forming opportunistic bacterial infections as they have a varied arsenal of antimicrobial molecules including organic acids, enzymes, surfactants and bacteriocins. Interference with biofilm occurs at different levels including attachment, quorum sensing, pathogen maintenance and disturbance of structural integrity. Another feature of probiotic species is that they compete effectively with other bacteria for the same ecological niche, and thereby prevent colonization by potential pathogens [155,156,157].

Several strains of the popular probiotic dietary supplement *Lactobacillus acidophilus* show anti-biofilm activity, and therefore are effective agents against *S. aureus*, including that produced by MRSA. Additionally, attachment, growth and formation of *S. aureus* biofilm is disturbed by *Lactobacillus plantarum*, *Limosilactobacillus fermentum* and *Pediococcus acidilactici*, each of which inhabits the human digestive tract. Among other probiotics with a potent activity towards bacterial biofilm are *Bifidobacterium lactis*, *B. longum*, *Lactobacillus brevis*, *L. casei*, *L. delbrueckii*, *L. fermentum*, *L. pentosus*, *L. rhamnosus*, *L. salivarius*, *L. sporogenes*, *Streptococcus oralis* and *S. salivarius.* Of these, *L. brevis* and *L. plantarum* were effective against *S. aureus* biofilm *in vitro*. Additionally, *in vivo* trials showed a protective effect of using *L. fermentum* to treat biofilm. Probiotics can be exploited for both prevention and treatment, but further research is needed to optimize efficacy [155,156,157,158,159,160,161].

*Rhamnolipids:* A number of alternative agents are being explored for their potential to treat biofilm (Table 1), primarily those formed by MRSA. Rhamnolipids are naturally occurring glycolipid biosurfactants that are produced predominantly by *P. aeruginosa*. They are harmless to humans and may thus be used in prescription medicines. This feature makes them an attractive candidate therapy for biofilm. Efficacy varies depending on differences in environmental conditions and in nutrient source and level [134,135,136]. In one study, rhamnolipid treatment removed 89% of biofilm attached to a skimmed milk-based agar substrate, but only 35% grown on nutrient medium, due to differing proportions of carbohydrate [162]. Rhamnolipids can disrupt biofilm in combination with caprylic acid and sophorolipids [163,164]. Mono-rhamnolipids have a bacteriostatic effect towards biofilm, while di-rhamnolipids show bactericidal properties [165]. Not only can formation of biofilm be prevented at low concentrations of caprylic acid, mature biofilm [166].

*Photodynamic therapy (PDT):* Established over a century ago, its common use developed only recently in response to heightened antibiotic resistance rates. PDT involves non-toxic photosensitizers whose activity is accelerated in the presence of oxygen, which can cause oxidative stress and cytotoxicity. Furthermore, activation takes place in the absence of oxygen through photoinactivation against anaerobic bacteria. The antibacterial mechanism is to target cell membrane, bacterial DNA, or enzymes [167].

This may be used to treat dental infections via oxidative damage of biofilm. Applying a low-power laser and photosensitizer in tandem is more beneficial to prevention of oral inflammation than to the detoxification of implant surfaces [168,169]. Combination therapy with antiseptics may boost PDT efficacy [170]. Successful attempts were made using photoditazine, fotoenticine and methylene blue to treat biofilm of *S. mutans*, *P aeruginosa* and MRSA [171,172]. In another *in vitro* study, synergism between antibiotics, indocyanine green and EDTA mediated PDT, which enhanced eradication of biofilm in MRSA-related infection [173]. PDT is considered as an alternative treatment for biofilm, specifically when it is combined with antibiotics or other inhibitors such as an efflux pump inhibitor or quorum sensing inhibitor. However, more *in vitro* and *in vivo* trials are needed [167].

*Nanoparticles and nanomaterials:* These have recently improved as an alternative method for biofilm treatment. Various classes of nanomaterial are used including carbon-based nanomaterials, polymeric nanoparticles, nano emulsions, nanocomposites, lipid nanoparticles and metallic oxide nanoparticles. Another, “smart nanomaterial”, has the potential to regulate drug release and alter its characteristics. Nanoparticles can deliver drugs to the site of infection. In addition, their simple preparation and flexible chemical formulation makes them a potential delivery tool for biofilm therapy. Nano-attapulgite, nano-TiO_2_, nano-Ag and SiO_2_, to name but a few, have shown antimicrobial effects when incorporated in food products [79,140,174,175].

Magnetic responsive nanomaterials are commonly used in magnetic resonance imaging. Activated by rising temperature, they can disperse cells embedded within biofilm. Recently, selenium and iron oxide nanoparticles in Galinstan (a gallium-indium-tin alloy that is liquid at room temperature) showed good anti-biofilm activity [176,177]. Nanomaterials that are responsive to light (e.g., DNase–AuNCs), pH (e.g., chitosan) or enzymes (e.g., micelles) exhibit antibiofilm activity through dispersing encapsulated bacteria, weakening biofilm matrix and reducing biofilm mass, respectively [178,179,180,181,182].

When applying nanomaterials a few factors should be considered. Firstly, translating *in vitro* trials to *in vivo* conditions may be challenging due to interaction with bacteria in the host body. The second point is insufficient knowledge of nanoparticle toxicity. Additionally, producing low-cost products and boosting efficiencies [79]. Regarding cytotoxicity, nanoparticles are responsible for various bioeffects including oxidative stress and autophagy [183]. For nanomaterials, it is the cell type, size and composition that determine the level of cytotoxicity and hence the fate of the cell [184].

*Bacteriophages:* Recently, bacteriophages were introduced as another potential approach. They may be described simply as viruses that can infect bacteria. Lytic phages, which kill the target cell through their replication, are well suited to therapeutic applications. Their small size allows permeation of the biofilm matrix. Additionally, they produce degradative enzymes that attack the ECM. In contrast to antibiotics, the efficacies of which are higher against planktonic cells, bacteriophages are more effective against bacteria within biofilm mass [185]. High specificity and low risk of resistance are further advantages of bacteriophage therapy [186].

Applications of phage therapy to biofilm treatment include phage-derived enzymes, modified phages, phage cocktails and combining phages with antibiotics. Careful attention should be paid to the specific characteristics of phages, such as their diffusion, penetration, and propagation [26,187]. Phage-derived lysin and depolymerase enzymes are introduced by lytic phages. LysCSA13, which is an *S. aureus* virulent bacteriophage CSA13 endolysin, under certain circumstances shows high antimicrobial activity against *S. aureus* [26,188]. Other bacteriophage lysins, such as CHAP(K), lysH5, phi11 and lysK, also show impressive anti-*S. aureus* properties [189,190,191]. Promising *in vitro* and *in vivo* results were attained when applying Csl2 against *S. suis* in zebrafish [192], as well as from testing the depolymerase phages Dpo7 and Dpo42 on *Staphylococcus spp.* and *Escherichia coli*, respectively [193,194].

Experimental use of the second type of bacteriophage against biofilm, genetically modified phage, has been highly successful. Examples are the *T7 E. coli* and modified *ΦEf11 E. faecalis* phages. The former is a phage that acts by expressing hydrolase, which achieved a more than 99% elimination rate [195,196]. Finally, combining phage therapy with antibiotics is a novel approach with higher efficacy compared to applying either treatment on its own. This is attributed to phage-antibiotic synergy, a phenomenon in which phage virulence is enhanced by exposure to a sub-lethal dose of antibiotic [197,198]. Studies using *Sb-1 S. aureus* and *T4* phage showed a synergistic effect on antibiotic efficacy against biofilm [199,200].

*Antimicrobial peptides:* AMPs are natural or synthetic oligopeptides that form part of the innate immune response of different organisms, and which have a wide range of inhibitory effects. Several antimicrobial peptides have been explored as novel treatment strategies. The twin public health challenges of biofilm-related infections and increasing prevalence of antibiotic resistance have led to the application of endogenous AMPs and antibodies that can each play a role in both treatment and prevention. AMPs show antibacterial activities through various mechanisms including interfering with bacterial cell signaling, destroying the cell membrane, and interrupting the bacterial alarmone system [201,202].

One of the first developed anti-biofilm peptides, human cathelicidin LL-37, has an ability to target preformed biofilm. Good activity was reported against biofilms of both Gram-positive and Gram-negative bacteria at one-twentieth of its MIC [203]. Moreover, modified LL-37 peptides showed high efficiencies against biofilm formation by *P. aeruginosa* [204]. Other LL-37 derivatives such as P60.4AC and P10 underwent satisfactory *in vitro* trials against multidrug-resistant *S. aureus*. Similarly, D-LL-37 was highly active against formation of biofilm and bacterial attachment by *P. aeruginosa* [205,206]. In one successful attempt to control MRSA, applying a cationic peptide lowered MIC values by two-fold [207]. Determining the suitability of each of these products to treat biofilm requires various considerations to be evaluated. From a therapeutic aspect, the extent of any cytotoxic damage should be recognized. AMPs can engender toxicity through pore formation, apoptosis, and necrosis [208].

In conclusion, applying anti-biofilm peptides, either natural or synthetic, has both advantages and disadvantages. The latter include increased manufacturing cost due to the long chain of peptides and complexity, high toxicities, and their susceptibility to host proteases. Modifications performed on peptides can ameliorate these development hurdles. On the other hand, the anti-biofilm activity of AMPs makes them an attractive choice as an alternative treatment. This is especially true if they can boost the efficiency of an antibiotic at a lower dose compared to single antibiotic therapy only [209].

There are yet further strategies used to combat biofilm infections, for which major investment is needed to underpin discovery and testing (Table 1). A current focus is on repurposing available drugs such as the anti-rheumatic agent auranofin. Several chelators such as ethylenediamine tetraacetic acid, sulfhydryl compounds like dithiothreitol, and phytochemicals extracted from plants, including flavonoids and polyphenolic compounds, are all under investigation [101]. Additionally, UM-C162, a benzimidazole derivative, shows therapeutic promise by interrupting various *S. aureus* virulence factors including hemolysins, clumping factors and proteases [210].

**Table 1:** Properties of different anti-biofilm agents.

Anti-Biofilm Agent	Mechanism of Action	Level of Interruption	Advantages	Disadvantages	References
**Rhamnolipids**	Disrupt biofilm	Adhesion Maturation process	High surface activity Biodegradability Low toxicity	Limited production Increasing usage is a threat to synthetic surfactants	[162,211]
**Photodynamics**	Affect bacterial LPS, endotoxin and cell differentiation	Mature biofilm	Synergic effect Strong treatment	Thermal damage Antibacterial resistance Surface modification	[167]
**Nanoparticles**	Transport drugs	Adhesion and mature biofilm	Small size Higher surface area to volume ratio	Toxicity	[79]
**Bacteriophages**	Disrupt biofilm	Mature biofilm	Specific for targets Effective against resistant strains	Further studies required Potential threat to human health	[26]
**Antimicrobial peptides**	Increase permeability of cell membrane	All three phases	Less chance of resistance Strong antibacterial activity	Further *in vivo* verification required Synthesis and purification are challenging	[209]
**Antibodies**	Help innate immune system	Adhesion and mature biofilm	Produce vaccine Prevention therapy	Further studies required	[78]
**Phytochemicals**	Reduce cell adhesion and disperse biofilm	Mature biofilm and dispersal	Natural compounds Strong antimicrobial agents	Poor solubility in aqueous media Further *in vivo* verification required	[212,213]
**Chelators and Sulfhydryl Compounds**	Decrease bacterial interaction and decrease PIA/PNAG	Adhesion	Potent antibiotic activity	Cytotoxic and genotoxic effects	[214]
**Laser Therapy**	Oxidative stress and disrupt bacterial cell wall	Mature biofilm	Boost antibiotic efficacy	High temperature in host tissue Cellular damage Further studies required	[215,216]
**Enzymes**	Target ECM and cell wall and increase chemical reaction	Adhesion and mature biofilm	Harmless to humans	Potential for activating immune system Further studies required	[154,217]

## 4. Future Directions

In the last few years, in response to the increasing public health threat posed by antibiotic resistance, considerable advances have been made in developing anti-biofilm prevention and treatment measures that can be applied at the bedside. Further fundamental research is needed to identify and validate novel approaches against the key targets of AMR, notably MRSA and VRSA.

Most biofilm prevention strategies are predicated narrowly on vaccines that target surface antigens or on surface coating of bacteria with chemical compounds or antibiotics. Meanwhile, treatment targets are broader, spanning all steps of biofilm formation from adhesion to dispersal. Notable approaches are the use of nanoparticles, laser therapy, probiotics, bacteriophages, and antibodies, each of which has strengths and weaknesses. As their efficacies and specificities are different, care should be taken in considering the treatment most appropriate for a patient among those available.

## 5. Conclusions

The public health challenge of antibacterial resistance has escalated considerably over recent decades. Of all potentially pathogenic species of bacteria those that form biofilm show heightened resistance to antibiotics. Foremost among these is *S. aureus*, in particular MRSA and VRSA. Determination of MIC and MBEC facilitates improved treatment of *S. aureus* biofilm infections. Although current approaches to combination therapy, typically using an antibiotic alongside an anti-biofilm agent, can achieve successful patient outcomes, complete removal of biofilm remains extremely difficult. Ongoing research aims to develop better means to address this important clinical concern.

The four successive steps of biofilm formation—attachment/adhesion, multiplication, maturation, and detachment/dispersal—as well as the mechanism of genetic regulation of each, are targets of experimental strategies for biofilm prevention and treatment. While specificity, molecular mechanisms and matrix components are distinct to each species, the general principles by which these steps function is common to all biofilm-forming bacteria. Hence, the much-studied MRSA is utilized as a valuable tool to explore biofilm development by opportunistic pathogenic bacteria.

## Data Availability

Not applicable.
